# Supplementary material for: Real-time signal processing via chemical reactions for a microfluidic molecular communication system
Source: Nat Commun. 2023 Nov 8;14:7188. doi: 10.1038/s41467-023-42885-0 (PMC10632502; doi:10.1038/s41467-023-42885-0)
Supplement: Supplementary file 1 — Supplementary Information [file 41467_2023_42885_MOESM1_ESM.pdf]

SUPPLEMENTARY INFORMATION

Real-time signal processing via chemical reactions for a microfluidic molecular communication system

Vivien Walter†[1], Dadi Bi†[1], Ali Salehi-Reyhani[2,3], and Yansha Deng*[1]

[1]Department of Engineering, King's College London, London, WC2R 2LS, United Kingdom
[2]Department of Surgery and Cancer, Imperial College London, London, W12 0HS, United Kingdom
[3]Institute of Molecular Sciences and Engineering, Imperial College London, London, SW7 2AZ, United Kingdom

†These authors contributed equally to this work.
*Corresponding Author: yansha.deng@kcl.ac.uk.

Contents

Supplementary note 1. Mathematical notations **3**

Supplementary note 2. Bromothymol Blue **5**

2.1 Absorption spectrum . . . 5
2.2 Effective range for the measurement of the BTB concentration . . . 7
2.3 Absorbance-pH mathematical modelling . . . 8
2.4 Determination of BTB constants . . . 9

Supplementary note 3. Measurement of the reaction time **11**

Supplementary note 4. pH Calibration for triangular flow rate signals **13**

Supplementary note 5. Signal analysis **14**

5.1 Detection threshold . . . 14
5.2 Pulse detection . . . 15
5.3 Pulse characteristics . . . 16

Supplementary note 6. Microfluidic experiments **17**

6.1 Signal amplification and detection (Figs. 2c-e) . . . 17
6.2 Signal thresholding (Figs. 3c-e) . . . 18
6.3 Signal suppression (Figs. 3f, g) . . . 19
6.4 Message transmission (Fig. 4a) . . . 20
6.5 High speed and long distance communication (Figs. 4c, d) . . . 22
6.6 Waveform Design - Software control (Figs. 5a, c) . . . 24
6.7 Waveform Design - Geometry control (Figs. 5b, d) . . . 25
6.8 Bit error rate measurement (Fig. 6a) . . . 26
6.9 Bit interval optimisation (Figs. 6c, d) . . . 29

Supplementary figure **30**

Supplementary note 1. Mathematical notations

The mathematical symbols used in the paper are summarized in Supplementary Table 1, and the symbols describing and defining pulses are illustrated in Supplementary Figure 1.

Notation	Physical Meaning
	General Notation
L_{CH}	The length of the propagation tubing connecting the transmitter and receiver
$\mathrm{pH_r}$	Revised pH of the solution obtained via the absorbance measured by the spectrometer, after conversion using Supplementary Equation (7)
$\mathrm{pH_a}$	Actual pH of the solution for which $\mathrm{pH_r}$ is measured by the spectrometer
[X]	Concentration of the chemical X
	Solutions
Y	Signal carrier
P	Signal suppressor, such as $\mathrm{Y} + \mathrm{P} \longrightarrow \phi$
ThL	Signal thresholder, such as $\mathrm{Y} + \mathrm{ThL} \longrightarrow \phi$
Amp	Signal amplifier, such as $\mathrm{Y} + \mathrm{Amp} \longrightarrow \mathrm{Y} + \mathrm{O}$
O	Visible output signal generated from $\mathrm{Y} + \mathrm{Amp} \longrightarrow \mathrm{Y} + \mathrm{O}$
Sol	Solvent
	Chemicals
NaOH	Sodium hydroxide
HCl	Hydrochloric acid
PBS	Phosphate-Buffered Saline
BTB	Bromothymol Blue (general notation)
HBTB	Bromothymol Blue, acid form
$\mathrm{BTB^-}$	Bromothymol Blue, base form
	Pulse & Bits Definition
T_{b}	Bit interval
α	The duty cycle, i.e., the fraction of the bit interval where the pump is turned on to inject signals
T_{e}	The width of the transmitted pulse
T_{o}	The observation time, i.e., the time between the injection of input Y and the observation of output O at the spectrometer
T	The width of the pulse generated at the receiver
ΔT	The transition time of a generated pulse to go from one state to the other
T_{d}	The delay between the injection of Y and P in the software
L_{d}	The difference in path length between the inlets of Y and P

Supplementary Table 1: **Notation table.**

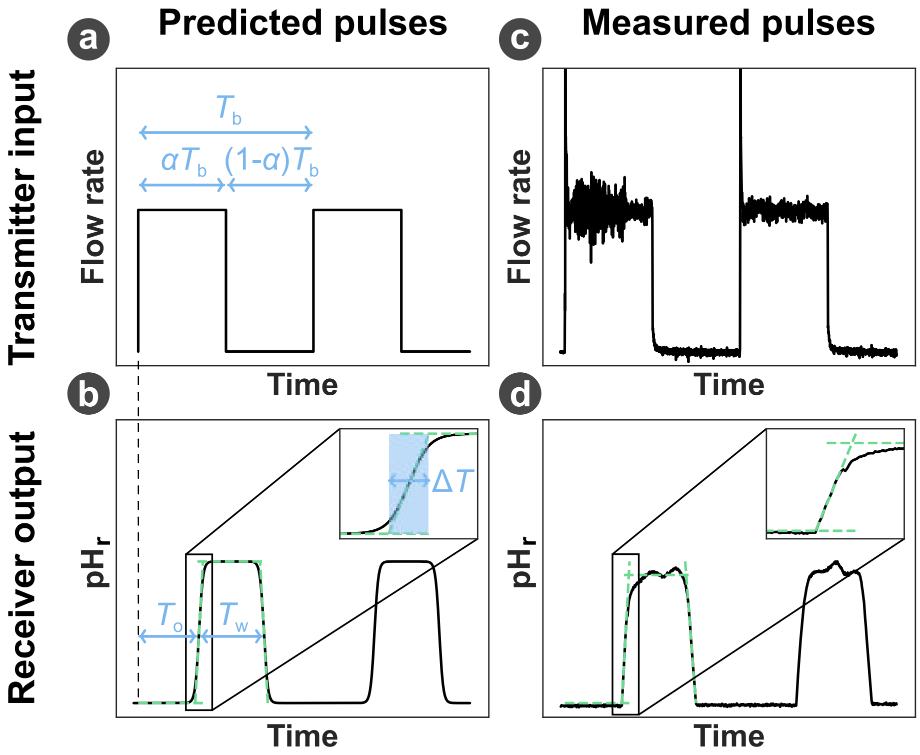

Supplementary Figure 1: **Pulse definition**. **(a-b)** Predicted theoretical pulses, based on (a) the transmission settings or (b) the prediction made on the effects of the propagation on the signal shape. The blue area highlights the transition period at the edge of the pulse. **(c-d)** Corresponding experimental measurements made (c) at the flow meter at the inlet of the transmitter or (d) by the spectrometer on the solution passing through the flow cell at the output of the receiver. All symbols presented in this figure are defined in Supplementary Table 1.

Supplementary note 2. Bromothymol Blue

2.1 Absorption spectrum

The color of a Bromothymol Blue (BTB) solution results from the presence of two molecular forms of BTB in this solution: an acid form with a yellow color, HBTB, and a base form with a blue color, BTB^-. The final color will be determined by the dominant species between these two forms, and can be expressed through the absorbance $A(\lambda)$ of the solution at the wavelength λ given by the Beer-Lambert equation:

$$A(\lambda) = l\{\epsilon_\mathrm{A}(\lambda)\,[\mathrm{HBTB}] + \epsilon_\mathrm{B}(\lambda)\,\left[\mathrm{BTB}^-\right]\}, \tag{1}$$

where [HBTB] and $[\mathrm{BTB}^-]$ are the concentrations of HBTB and BTB^- at steady state, l is the optical path length, and $\epsilon_\mathrm{A}(\lambda)$ and $\epsilon_\mathrm{B}(\lambda)$ are the molar attenuation coefficients of HBTB and BTB^- at wavelength λ, respectively. According to [1,2], the wavelengths at which HBTB and BTB^- reach their maximum absorption are 453 nm and 616 nm, respectively.

The ratio between $[\mathrm{BTB}^-]$ and [HBTB] inside a solution can be calculated from the pH of that solution using the equation

$$\frac{[\mathrm{BTB}^-]}{[\mathrm{HBTB}]} = K_\mathrm{C}10^{\mathrm{pH}}, \tag{2}$$

where K_C is the equilibrium constant of reaction

$$\mathrm{HBTB} \xleftrightarrow{\mathrm{pKa}\,=\,7.10} \mathrm{H}^+ + \mathrm{BTB}^-. \tag{3}$$

Due to this equilibrium between both forms, the values of [HBTB] and $[\mathrm{BTB}^-]$ are always dependent on each other, and the sum of [HBTB] and $[\mathrm{BTB}^-]$ is a constant, which is

$$[\mathrm{BTB}]_\mathrm{Tot} = [\mathrm{BTB}^-] + [\mathrm{HBTB}]. \tag{4}$$

With Supplementary Equations (2) and (4), Supplementary Equation (1) can be rewritten to express the absorbance as a function of the pH of the solution

$$A(\lambda) = l[\mathrm{BTB}]_\mathrm{Tot}\left\{\epsilon_\mathrm{B}(\lambda) + \frac{\epsilon_\mathrm{A}(\lambda) - \epsilon_\mathrm{B}(\lambda)}{1 + K_\mathrm{C}10^{\mathrm{pH}}}\right\}. \tag{5}$$

As l, ϵ_A, ϵ_B, and K_C are all constant, the color of a solution only depends on the total concentration of dye $[\mathrm{BTB}]_\mathrm{Tot}$ and the pH of the solution. Supplementary Figure 2a experimentally demonstrates that with an increase of the pH of the solution from 2 to 12 the solution color changed from yellow (acid form) to blue (base form), with a transition range appearing green (coexistence between acid and base forms). Experimental data also confirmed the wavelengths that reach the maximum absorbance reported in [1,2] (Supplementary Figure 2b).

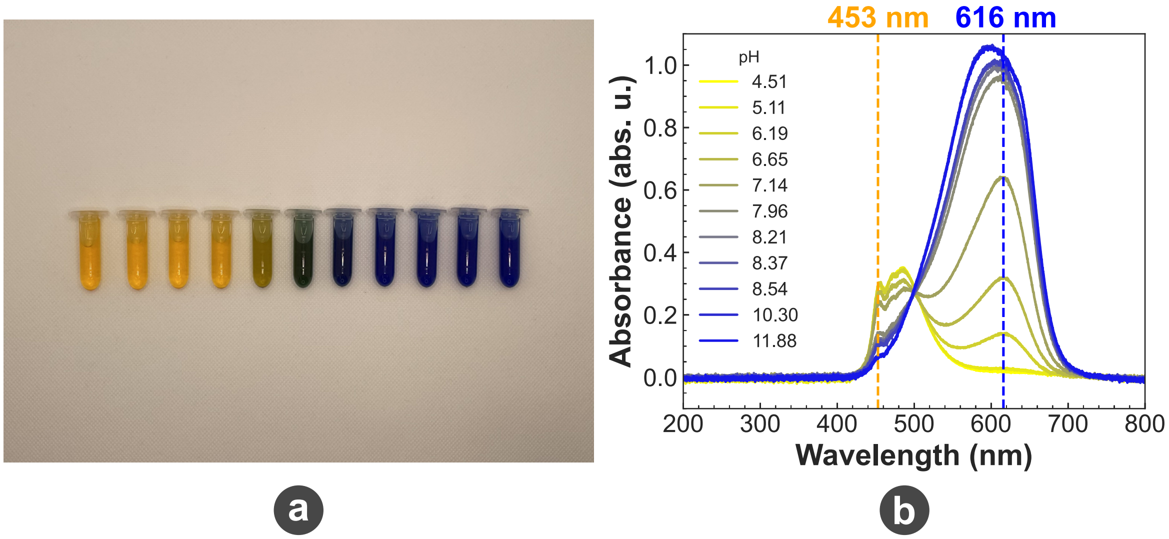

Supplementary Figure 2: **Spectrum of the BTB Solution**. **(a)** Evolution of the color of the BTB solution with the pH of the solution, ranging from pH 2 to pH 12 (from left to right). **(b)** Evolution of the absorbance spectrum of the BTB solution with the solution pH. The vertical dashed lines highlight the peak of maximum absorbance for the acid (453 nm) and base forms (613 nm) reported in [1, 2].

2.2 Effective range for the measurement of the BTB concentration

The application of Supplementary Equation (5) to the absorbance measurement in a solution requires a proper definition of the effective measurement range of BTB concentration. The absorbance measured by a UV-Vis spectrometer can be defined as

$$A = \log_{10}\left(\frac{I_0}{I}\right), \tag{6}$$

where I_0 is the light intensity collected by the spectrometer for a reference solution and I is the light intensity for the interested solution. In our experiments, the reference solution is a pure PBS solution.

From Supplementary Equation (6), when I is too low (high BTB concentration) or too high (low BTB concentration), a variation of I will lead to a small change in the absorbance, meaning the concentration change can be hardly detected by the spectrometer and leading to a nonlinear relationship between the BTB concentration and the absorbance. As a consequence, Supplementary Equations (1) and (5) are only valid for a given range of BTB concentrations. We measured this range experimentally by studying the evolution of the intensity collected by a spectrometer as a function of the total BTB concentration in solution $[\mathrm{BTB}]_{\mathrm{Tot}}$ (Supplementary Figure 3). By setting the intensity at 80% of the full resolution of the sensor for a pure PBS solution, the linear range was found as $[1 \times 10^{-5}, 2 \times 10^{-4}]$ mol L^{-1}.

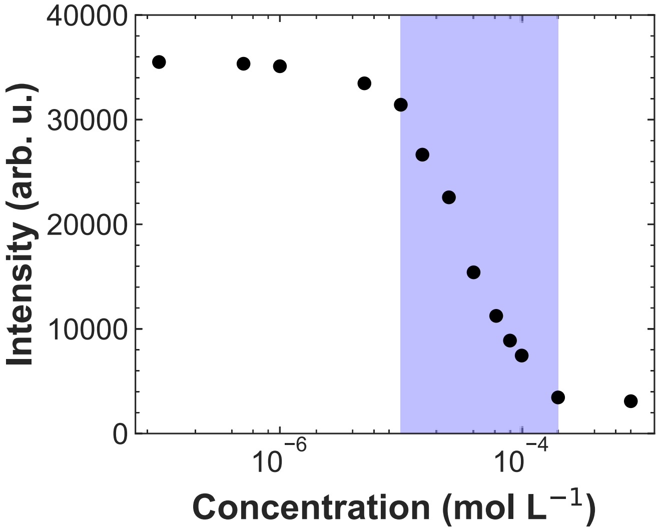

Supplementary Figure 3: **Effective range for the measurement of the BTB concentration.** The area highlighted in blue corresponds to the range in which the relation between the intensity and the concentration can be considered linear.

2.3 Absorbance-pH mathematical modelling

Although Supplementary Equation (5) correlates the absorbance of a solution with its pH, the $[\mathrm{BTB}]_{\mathrm{Tot}}$ is likely to vary not only from experiment to experiment, especially under a flow chemistry setup (e.g., our MIMIC platform), but also during a single experiment as it is impacted by the relative flow rate of the pump injecting BTB to the total output flow rate at all times.

To eliminate the impact of $[\mathrm{BTB}]_{\mathrm{Tot}}$ fluctuation, we consider the ratio of $A(616)$ to $A(453)$ and derive the expression of the revised pH of the solution $\mathrm{pH_r}$ as

$$\mathrm{pH_r} = \log_{10}\left\{\frac{\left(\frac{A(616)}{A(453)}\right)(\epsilon_{\mathrm{A}}(453)-\epsilon_{\mathrm{B}}(453))-(\epsilon_{\mathrm{A}}(616)-\epsilon_{\mathrm{B}}(616))}{K_{\mathrm{C}}\epsilon_{\mathrm{B}}(616)-K_{\mathrm{C}}\left(\frac{A(616)}{A(453)}\right)\epsilon_{\mathrm{B}}(453)}-\frac{1}{K_{\mathrm{C}}}\right\}. \tag{7}$$

It is clear that this equation removes the dependency of solution pH on both l and $[\mathrm{BTB}]_{\mathrm{Tot}}$, which effectively prevents the measurement of the absorbance from being impacted by fluctuations from the flow and enables an indirect measurement of the solution pH using a UV-Vis spectrometer.

2.4 Determination of BTB constants

The calculation of the revised pH of a solution pH_r via Supplementary Equation (7) requires to determine the parameters of $\epsilon_A(453)$, $\epsilon_A(616)$, $\epsilon_B(453)$, $\epsilon_B(616)$, and K_C. The reaction equilibrium constant K_C was taken from [1, 2], with a value equal to 7.9×10^{-8} mol L^{-1}.

The values of the molar attenuation coefficients $\epsilon_A(453)$, $\epsilon_A(616)$, $\epsilon_B(453)$, and $\epsilon_B(616)$ were measured experimentally through the measurement of the absorbance of solutions made at different BTB concentrations, ranging from 0.01 to 0.1 mmol L^{-1}. The pH of the solutions was set either to pH = 4.78 for the coefficients $\epsilon_A(453)$ and $\epsilon_A(616)$ of the acid form HBTB, or to pH = 10.88 for the coefficients $\epsilon_B(453)$ and $\epsilon_B(616)$ of the acid form BTB^-. Experimental data are shown in Supplementary Figure 4. For each molar attenuation coefficient, its value is calculated by fitting the experimental data to the Beer-Lambert equation

$$A = l\epsilon[\mathrm{BTB}]_{\mathrm{Tot}}, \tag{8}$$

with $l = 0.25$ cm taken from the geometry of the flow cell used for the measurement (Z-type, FIAlabs, USA). At the pH values used for both measurements, we assume that the concentration of the dominant form of BTB in the solution is equal to $[\mathrm{BTB}]_{\mathrm{Tot}}$, which can be revealed by Supplementary Equation (2). The fitting provides the following value for the parameters:

- $\epsilon_A(453) = 7370 \pm 50$ L mol^{-1}, $\epsilon_A(616) = 1290 \pm 10$ L mol^{-1},
- $\epsilon_B(453) = 240 \pm 20$ L mol^{-1}, $\epsilon_B(616) = 31800 \pm 800$ L mol^{-1}.

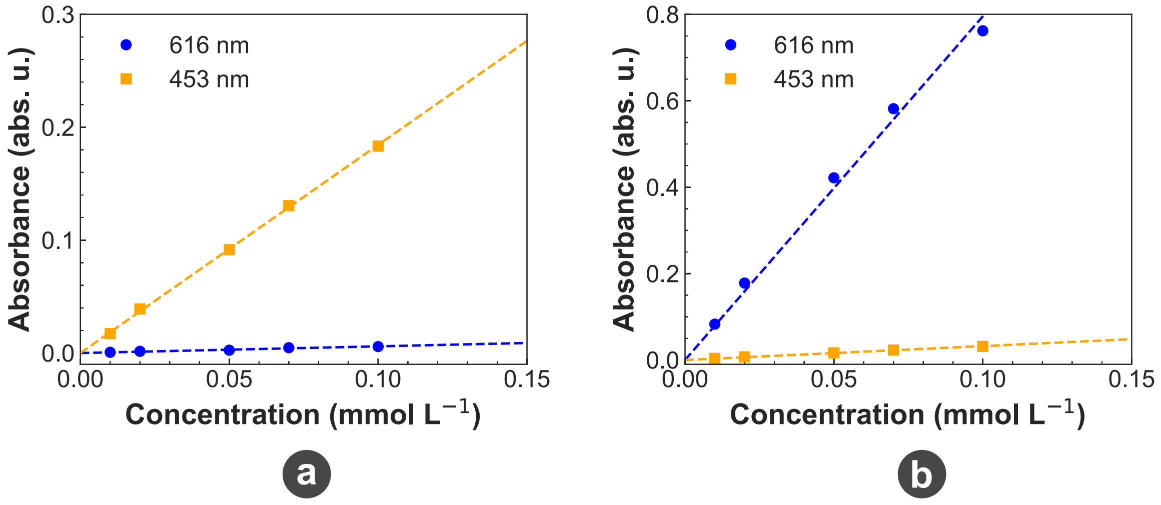

Supplementary Figure 4: **Determination of the molar attenuation coefficients**. Evolution of the absorbance of a BTB solution at **(a)** pH = 4.78 (acid) and **(b)** pH = 10.88 (base) with the total concentration of BTB $[BTB]_{Tot}$, measured at 453 and 616 nm. The dashed lines are the linear fitting via Supplementary Equation (8).

Supplementary note 3. Measurement of the reaction time

As discussed in the main text, the tubing length used in different parts of the MIMIC platform should be long enough to ensure that the chemical reaction starting at the inlet of a tubing is completed before the solution reaches the outlet of that same tubing. The chemical reaction is complete if the residence time T_r (the time of the solution going through the whole tubing) is larger or equal to the reaction time T_χ, which is

$$T_r \geq T_\chi. \tag{9}$$

The residence time T_r can be calculated as

$$T_r = \frac{L_r S}{Q_r}, \tag{10}$$

where L_r is the tubing length, S is the area of the cross-section, and Q_r is the flow rate of the solution.

When the reaction time T_χ is unknown, we can experimentally obtain it through Supplementary Equation (10) and measuring a quantity related to the reaction yield. In the example of the amplification reaction (equation (1) in the main text), the pH of

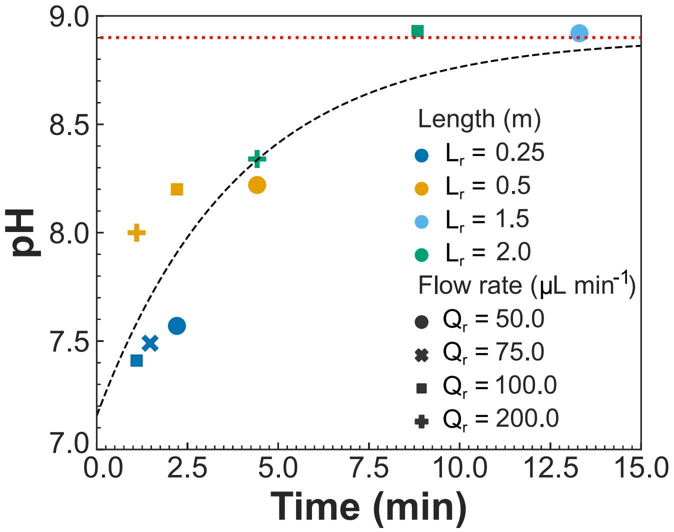

Supplementary Figure 5: **Reaction time**. Measurement of the reaction time of the reaction between Y (pH 10.8) and Amp through the evolution of the pH measured at the output of the microfluidic circuit. The different residence times were obtained by varying either the tubing length L_r or the flow rate Q_r. The dashed black line is the logarithmic fit used to determine the reaction time, and the dotted orange line is the expected pH of the O solution.

the solution is expected to start from the pH of the pure Amp solution (i.e., pH=5.3) and slowly increase upon reaction until it reaches the expected pH of the O solution (i.e., pH=8.9). Therefore, we measured the pH values at the outlet of the tubing for different residence times by modifying either the tubing length L_r or the flow rate Q_r. The results are shown in Supplementary Figure 5.

As the pH only slowly converges toward the final value of 8.9, we set the value of the reaction time T_χ as the value of T_r when 95 % of the reaction has been completed, corresponding to a pH of 8.72. Using this method and the experimental results, we measured a reaction time in our tubing of $T_\chi = 10$ min.

Supplementary note 4. pH Calibration for triangular flow rate signals

In order to obtain the pH profile of the transmitted signals (i.e., the pH profile at X_1 in Fig. 2 and X_2 in Fig. 3) when the input signal Y follows a triangular profile in flow rate while measuring the receiver output at the flow cell, we calibrated and measured the pH values of the mix of different volume ratios of solution Y to solvent in traditional glassware (Supplementary Figure 6). If these solutions are injected and then propagated in the tubing with a constant cross-section area, their volume ratio is equivalent to their flow rate ratio. Therefore, knowing the ratio between the flow rates of the two solutions allows us to calculate the pH of the corresponding mixed solution propagating in the tubing. The experimental values reported in Supplementary Figure 6 were fitted with a simple logarithmic model to obtain a calibration function that estimates the pH of any flow rate ratio, which is

$$\mathrm{pH} = k_1 \log_{10}\left(\frac{V_\mathrm{Y}}{V_\mathrm{Sol}} + k_2\right) + k_3, \tag{11}$$

where V_Y and V_Sol are the volumes of solution Y and Sol used for mixing, and the fitting parameters are $k_1 = 1.26 \pm 0.06$, $k_2 = 1.3 \times 10^{-4} \pm 5 \times 10^{-5}$, and $k_3 = 12.46 \pm 0.03$.

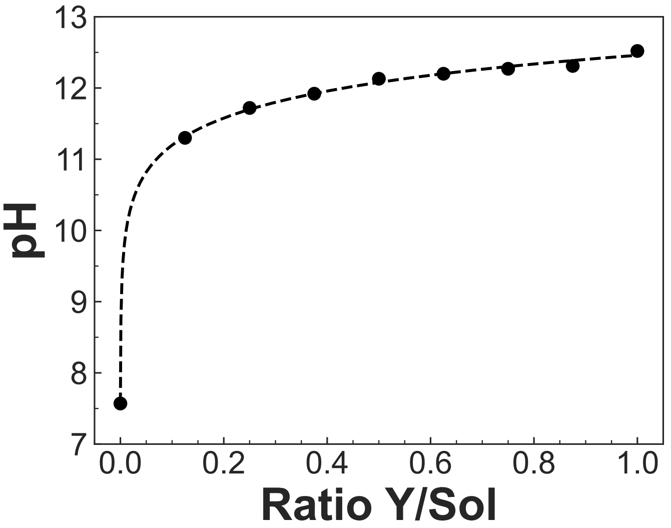

Supplementary Figure 6: **pH calibration**. Calibration of the pH value of a solution obtained through mixing a Y solution with the solvent solution Sol at different volume ratios. Experimental data were fitted with a logarithmic model.

Supplementary note 5. Signal analysis

The time-varying pH data collected by the spectrometer at the receiver are analysed to detect and characterize the pulses.

5.1 Detection threshold

To determine the pulse detection threshold, we first studied the distribution of the pH values collected by the spectrometer. From Supplementary Figure 7a, the pH data mainly distributed within two regions, and we calculated the mean values of these two regions, named μ_{off} and μ_{on}. The detection threshold pH_τ is then set at -3 dB of the difference between μ_{off} and μ_{on} (Supplementary Figure 7), which is

$$\text{pH}_\tau = \mu_{\text{off}} + \frac{(\mu_{\text{on}} - \mu_{\text{off}})}{\sqrt{2}}. \quad (12)$$

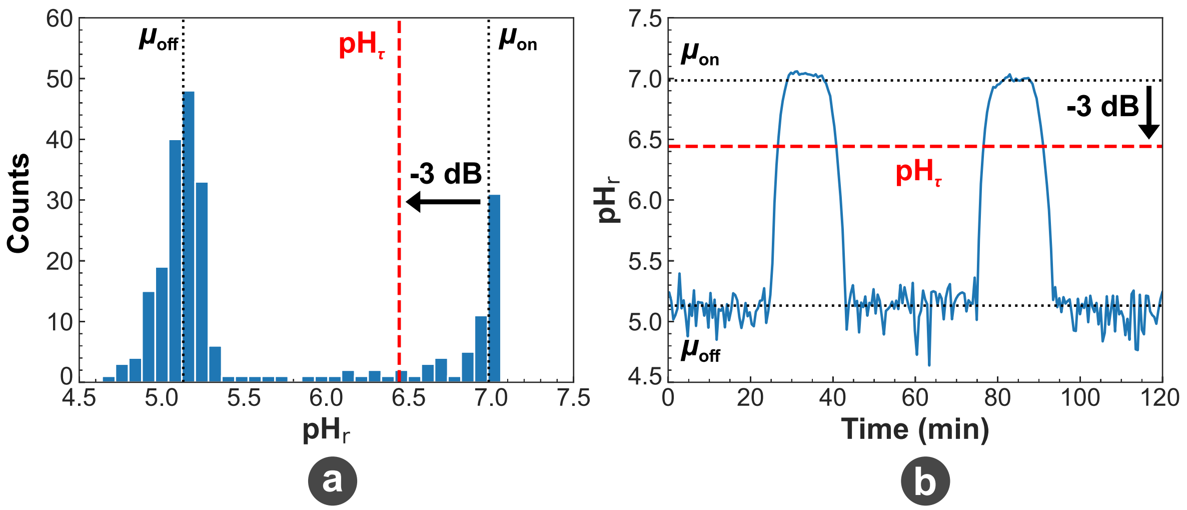

Supplementary Figure 7: **Detection threshold**. **(a)** Distribution of the pH measurements in the considered time frame. The detection threshold pH_τ is defined as -3 dB of the difference between μ_{off} and μ_{on}. **(b)** Signal used to obtain the distribution shown in (a), with the corresponding quantities measured on the distribution.

5.2 Pulse detection

To localise the pulses, we first detect the pulse edges through

$$pH_{signed}(t_i) = (pH_r(t_i) - pH_\tau) \times (pH_r(t_{i-1}) - pH_\tau) \,, \tag{13}$$

where $pH_r(t_i)$ and $pH_r(t_{i-1})$ are the pH measurement sampled at time t_i and t_{i-1}, respectively. When $pH_{signed}(t_i) < 0$, a pulse edge is detected at time t_i. Then, the pulses can be identified based on these pulse edges (Supplementary Figure 8).

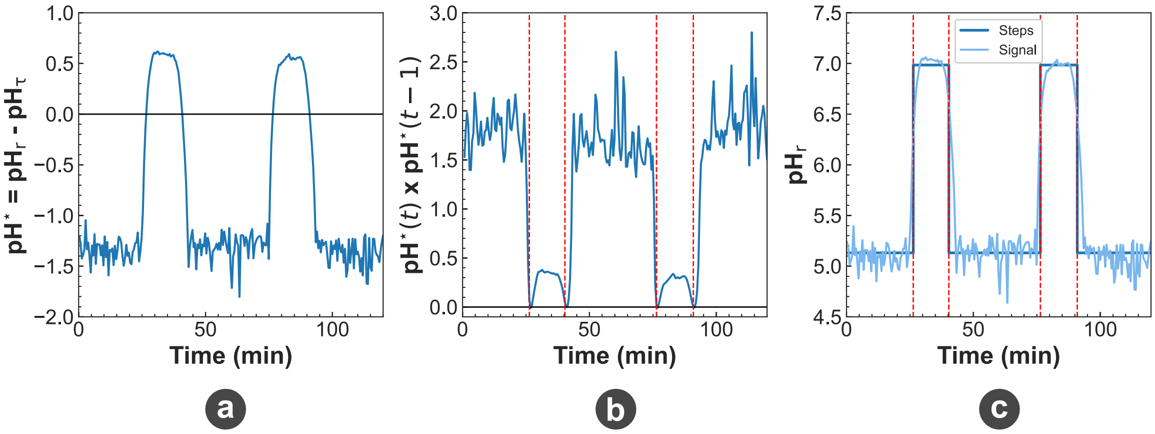

Supplementary Figure 8: **Pulse detection**. Presentation of the successive calculations used to detect the pulse edge times and thus the pulses from measured signals. **(a)** Measurement of the value of $pH^\star$, corresponding to the value of the revised pH_r to which the detection threshold has been subtracted. **(b)** Multiplication of each value of $pH^\star$ by the previous value in time, followed by detection of the time at which this product is a negative value. The dashed red lines represent the pulse edge times measured using Supplementary Equation (13). **(c)** Reconstruction of the pulses using the times detected in (b) and the average values between two consecutive times.

5.3 Pulse characteristics

The observation time T_o and demodulated signal width T_w can be directly calculated through the times of pulse edges. For the transition time ΔT, we first fitted the pulse edge with a Sigmoid function and then calculated ΔT from the fitted increase rate as well as the fitted low and high pH values (Supplementary Figure 9).

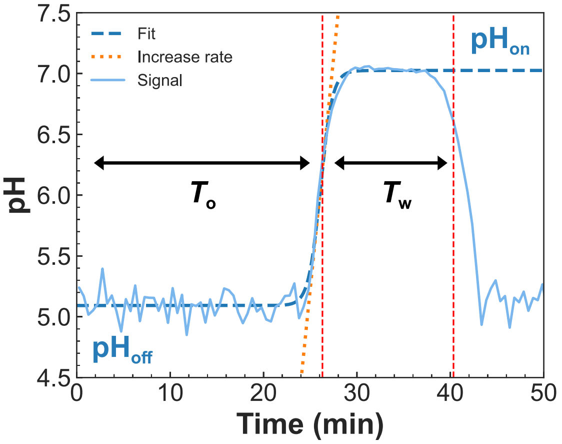

Supplementary Figure 9: **Pulse characteristics**. Illustration of the calculation of observation time T_o, demodulated signal width T_w, and transition time ΔT for a pulse. The dashed red lines represent the pulse edge times measured using Supplementary Equation (13), and the dashed blue line is the Sigmoid fitting between the plateaux defined as pH_off and pH_on of the first pulse edge.

Supplementary note 6. Microfluidic experiments

6.1 Signal amplification and detection (Figs. 2c-e)

Experiment description. Investigation of the reaction between Y and Amp.
Experimental setup. Simplified architecture of the MIMIC platform, shown in Fig. 2b: two inlets at the transmitter, (1) the Y pump and (2) the Sol pump, and two inlets at the receiver, (3) the inlet connected to the output of the transmitter and (4) the Amp pump. Distance between T junction of Y + Amp and the UV-Vis spectrometer: 2 m.
Solution injection. The Y injection flow rate $Q_{\mathrm{Y}}(t)$ followed a triangular distribution in equation (7) in the Methods of the main text. The Sol injection flow rate $Q_{\mathrm{Sol}}(t)$ was adjusted accordingly to maintain a 48 µL min^{-1} constant flow rate at the output of the transmitter. The solutions' pH and injection flow rates are summarized in Supplementary Table 2.

Solution	Y	Sol	Amp
pH	10.8	7.58	5.3
Flow rate (µL min^{-1})	$Q_{\mathrm{Y}}(t)$	$48 - Q_{\mathrm{Y}}(t)$	16

Supplementary Table 2: **Signal amplification and detection.** Composition and injection flow rate of the different solutions injected for the experiment in Figs. 2c-e. Three concentrations of BTB were used in these experiments: 2×10^{-4}, 5×10^{-5}, and 2×10^{-5} mol L^{-1}.

6.2 Signal thresholding (Figs. 3c-e)

Experiment description. Investigation of the reaction between Y and ThL.
Experimental setup. Simplified architecture of the MIMIC platform, shown in Fig. 3b: two inlets at the transmitter, (1) the Y pump and (2) the Sol pump, and three inlets at the receiver, (3) the inlet connected to the output of the transmitter, (4) the ThL pump, and (5) the Amp pump. The Amp solution was used in this experiment to allow us to visualise the effect of the ThL solution on the Y signal. Distance between the Y + Amp T junction and the UV-Vis spectrometer: 2 m.
Solution injection. The Y injection flow rate $Q_{\mathrm{Y}}(t)$ followed a triangular distribution in equation (7) in the Methods of the main text. The Sol injection flow rate $Q_{\mathrm{Sol}}(t)$ was adjusted accordingly to maintain a 48 µL min^{-1} constant flow rate at the output of the transmitter. The solutions' pH and injection flow rates are summarized in Supplementary Table 3.

Solution	Y	Sol	ThL	Amp
pH	10.8	7.58	various[a]	5.3
Flow rate (µL min^{-1})	$Q_{\mathrm{Y}}(t)$	$48 - Q_{\mathrm{Y}}(t)$	16	16

[a] Three different pH values were used for ThL: 1.2, 1.6, and 7.58.

Supplementary Table 3: **Signal thresholding.** Composition and injection flow rate of the different solutions injected for the experiment in Figs. 3c-e. When the pH of ThL solution was 7.58, it was replaced by a solvent solution.

6.3 Signal suppression (Figs. 3f, g)

Experiment description. Investigation of the reaction between Y and P.
Experimental setup. Full 5-pump version of the MIMIC platform, as illustrated in Fig. 1a. Distance between the transmitter and the receiver: 2 m. Distance between the final T-junction at the receiver and the UV-Vis spectrometer: 2 m.
Solution injection. The experiment was conducted in 3 separate phases, and the solutions' pH, solution injection, and flow rate are summarized in Supplementary Table 4.

- **Phase 1**: Cleaning and baseline, 30-min duration. Running pump Sol, pump ThL, and pump Amp to remove signal Y from the platform and provide a baseline for the experiment.
- **Phase 2**: Signal injection, 15-min duration. Running pump Y, pump Sol, pump ThL, and pump Amp.
- **Phase 3**: Signal and suppressor injection, lasting until the experiment was terminated. Running pump Y, pump P, pump Sol, pump ThL, and pump Amp.

Solution		Y	P	Sol	ThL	Amp
pH		12.5	1.5	7.58	1.6	5.3
Flow rate (μL min^{-1})	Phase 1: Cleaning & baseline	0	0	48	16	16
	Phase 2: Signal injection	12	0	36	16	16
	Phase 3: Signal & suppressor injection	12	12	24	16	16

Supplementary Table 4: Composition and injection flow rate of the different solutions injected for the experiment in Figs. 3f, g. To output a constant flow rate at the transmitter during the whole experiment, the flow rate of the Sol pump follows $Q_{\text{Sol}}(t) = 48 - (Q_{\text{Y}}(t) + Q_{\text{P}}(t))$.

6.4 Message transmission (Fig. 4a)

Experiment description. Transmission of the message "Hi".
Experimental setup. Full 6-pump version of the MIMIC platform, illustrated in Fig. 1a but with the transmitter modification presented in Fig. 5b. Distance between the transmitter and the receiver: 2 m. Distance between the final T-junction at the receiver and the UV-Vis spectrometer: 2 m.
Solution injection. The experiment was conducted in 4 phases, and the solution's pH, solution injection, and flow rates are summarized in Supplementary Table 5.

- **Phase 1**: Cleaning and baseline, 30-min duration. Running two pumps Sol, pump ThL, and pump Amp to remove signal Y from the platform and provide a baseline for the experiment.
- **Phase 2**: Transmission initialisation. Running the pumps to transmit two bit-1, indicating the start of a message.
- **Phase 3**: Message transmission. Running the pumps to transmit bit sequence "10010001101001" (ASCII) for the message "Hi".
- **Phase 4**: Signal collection, 60-min duration. Running two pumps Sol, pump ThL, and pump Amp to ensure the reception of all transmitted bits.

Specifically, the injection of bit-0 and bit-1 was performed as follows:

- Bit-0 transmission: For the whole bit interval $T_\mathrm{b} = 50$ min, only running two pumps Sol, pump ThL, and pump Amp.
- Bit-1 transmission:
  - From the beginning of the bit interval until time $\alpha T_\mathrm{b} = 30$ min (α is the duty cycle), running all the pumps.
  - For the remaining $(1 - \alpha)T_\mathrm{b} = 20$ min, only running two pumps Sol, pump ThL, and pump Amp as the guard interval.

Solution			Y	P	Sol Y	Sol P	ThL	Amp
pH			12.5	1.5	7.58	7.58	1.6	5.3
Flow rate (μL min^{-1})	Phase 1: Cleaning & baseline		0	0	24	24	16	16
	Phase 2&3: Bit transmission	Bit-0	0	0	24	24	16	16
		Bit-1 duty cycle	12	12	12	12	16	16
		Bit-1 guard interval	0	0	24	24	16	16
	Phase 4: Signal collection		0	0	24	24	16	16

Supplementary Table 5: **Message transmission.** Composition and injection flow rate of the different solutions injected for the experiment in Fig. 4a. To output a constant flow rate at the transmitter during the whole experiment, the flow rates of the two Sol pumps follow $Q_{\mathrm{SolY}}(t) = 24 - Q_{\mathrm{Y}}(t)$ and $Q_{\mathrm{SolP}}(t) = 24 - Q_{\mathrm{P}}(t)$, respectively.

6.5 High speed and long distance communication (Figs. 4c, d)

Experiment description. Assessment of the capability of the MIMIC platform to communicate at high speed and over long distances, by sending bit-1 repeatedly.
Experimental setup. Full 6-pump version of the MIMIC platform, illustrated in Fig. 1a but with the transmitter modification presented in Fig. 5b. Distance between the transmitter and the receiver: 25 m. Distance between the final T-junction at the receiver and the UV-Vis spectrometer: 2 m. In this experiment, only sequences of bit-1 were transmitted.
Solution injection. The experiment was conducted in 3 phases and repeated four times using different speed factor N. The values of N, communication settings, and injection flow rates are summarized in Supplementary Table 6. The pH values of the solutions can be found in Supplementary Table 5.

- **Phase 1:** Cleaning and baseline. Running two pumps Sol, pump ThL, and pump Amp to remove signal Y from the platform and provide a baseline for the experiment.
- **Phase 2:** Bit sequence transmission, during which bit-1 are repeatedly transmitted.
- **Phase 3**: Signal collection. Running two pumps Sol, pump ThL, and pump Amp to ensure the reception of all transmitted bits.

Speed factor N		Unit	1	2	4	8
Data rate		bit min^{-1}	0.02	0.04	0.08	0.16
Bit interval T_b		min	50	25	12.5	6.25
Injection duration αT_b		min	30	15	7.5	3.75
Y-P injection delay[a] T_d		min	20	10	5	2.5
Flow rate	Y	µL min^{-1}	12	24	48	96
	P		12	24	48	96
	ThL		16	32	64	128
	Amp		16	32	64	128
	Sol Y & Sol P [b]		12	24	48	96
	Sol Y & Sol P [c]		24	48	96	192
	Receiver output		80	160	320	640

[a] The delay was fully set via modification of the platform geometry.
[b] Injection flow rate when the corresponding Y or P pump is also injected.
[c] Injection flow rate when the corresponding Y or P pump is not injected.

Supplementary Table 6: Communication settings and injection flow rate of the different solutions injected for the experiments in Figs. 4c, d.

Supplementary results. The impact of the flow rate on the demodulated signal was investigated in terms of the observation time T_o, demodulated signal width T_w, and

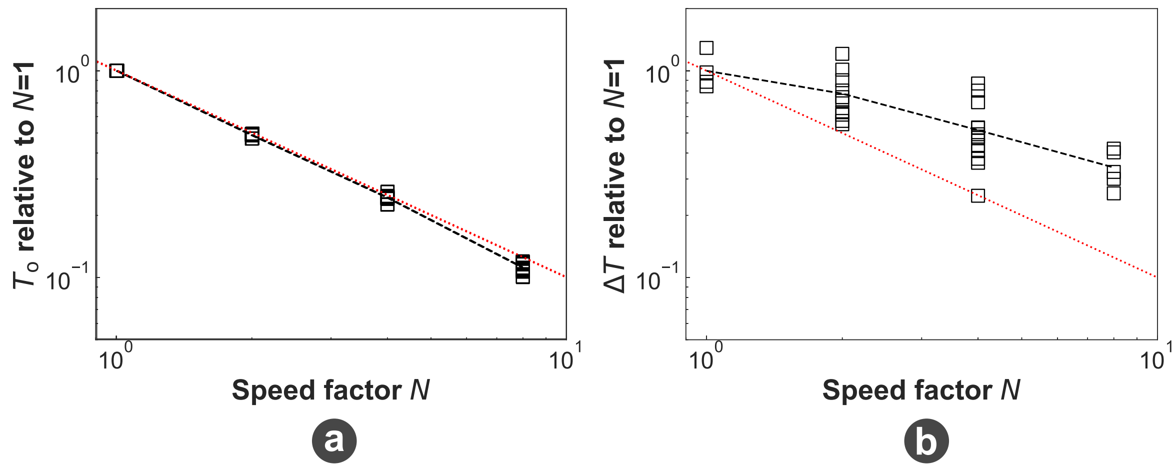

Supplementary Figure 10: **High speed effect on the pulse shape**. Evolution of the impact of the speed factor on the **(a)** observation time T_o and the **(b)** transition time ΔT of the demodulated pulse. The red dashed line represents the theoretical proportional $1/N$ evolution. The evolution of T_w is provided in Fig. 4c of the main text.

demodulated signal transition time ΔT (illustrated in Supplementary Figure 1). Both T_o (Supplementary Figure 10a) and T_w (shown in the main text, Fig. 4d) display a clear proportionality with N. For ΔT, a similar relationship with N was only partially observed (Supplementary Figure 10b). This is due to the fact that the transition time ΔT is affected not only by the advection but also by the chemical diffusion [3]. However, that effect on the shape of the signals does not prevent our platform from communicating at the highest achievable speed, as demonstrated in Fig. 4c.

6.6 Waveform Design - Software control (Figs. 5a, c)

Experiment description. Control the width of the transmitted signal via Python software.

Experimental setup. Full 5-pump version of the MIMIC platform, as illustrated in Fig. 1a. Tubing length: $L_1 = L_2 = 5$ cm. Distance between the transmitter and the receiver: 2 m. Distance between the final T-junction at the receiver and the UV-Vis spectrometer: 2 m.

Solution injection. The experiment was conducted in the same 3 separate phases as the Signal suppression experiment 6.3, with the same solutions presented in Supplementary Table 4. The duration of the second phase used in these experiments was 10, 20, 30, 40, and 50 min.

6.7 Waveform Design - Geometry control (Figs. 5b, d)

Experiment description. Control the width of the transmitted signal by adjusting the microfluidic geometry.

Experimental setup. Full 6-pump version of the MIMIC platform, as illustrated in Fig. 1a but with the transmitter design shown in Fig. 5b. Tubing length: $L_1 = 5$ cm. Different tubing lengths L_2 were used: 51, 105, 159, 213, and 267 cm, resulting in different path differences $L_\mathrm{d} = L_2 - L_1$ of 46, 100, 154, 208, and 262 cm. Distance between the transmitter and the receiver: 2 m. Distance between the final T-junction at the receiver and the UV-Vis spectrometer: 2 m.

Solution injection. The experiment was conducted in 2 phases, and the solutions' pH, solution injection, and flow rates are summarized in Supplementary Table 7.

- **Phase 1:** Cleaning and baseline, 30-min duration. Running two pumps Sol, pump ThL, and pump Amp to remove signal Y from the platform and provide a baseline for the experiment.

- **Phase 2:** Signal and suppressor injection, lasting until the experiment was terminated. Running pump Y, pump P, pump Sol, pump ThL, and pump Amp.

Solution		Y	P	Sol Y	Sol P	ThL	Amp
pH		12.5	1.5	7.58	7.58	1.6	5.3
Flow rate (µL min^{-1})	Phase 1: Cleaning & baseline	0	0	24	24	16	16
	Phase 2: Y & P injection	12	12	12	12	16	16

Supplementary Table 7: **Waveform Design.** Composition and injection flow rate of the different solutions injected for the experiment in Figs. 5b, d. To output a constant flow rate at the transmitter during the whole experiment, the flow rate of the two Sol pumps follow $Q_\mathrm{SolY}(t) = 24 - Q_\mathrm{Y}(t)$ and $Q_\mathrm{SolP}(t) = 24 - Q_\mathrm{P}(t)$.

6.8 Bit error rate measurement (Fig. 6a)

Experiment description. Measurement of the bit error rate (BER) under different transmission settings.
Experimental setup. Full 6-pump version of the MIMIC platform, as illustrated in Fig. 1a but with the transmitter modification presented in Fig. 5b. Tubing length: $L_1 = 5$ cm and $L_2 = 105$ cm. Distance between the transmitter and the receiver: 25 m. Distance between the final T-junction at the receiver and the UV-Vis spectrometer: 2 m.
Solution injection. The experiment was conducted in 3 phases and repeated three times for different duty cycles α with the same 100 bits transmitted. The solutions' pH, solution injection, and flow rates are summarized in Supplementary Table 8.

- **Phase 1:** Cleaning and baseline, 3.75-min duration. Running two pumps Sol, pump ThL, and pump Amp to remove signal Y from the platform and provide a baseline for the experiment.

- **Phase 2:** Message transmission. Running the pumps to transmit three subsequences for a total of 100 bits.
  - The first subsequences: "0001001110001001000110100001010";
  - The second subsequences: "11011000001110100011000011101110011001010 -0000110";
  - The third subsequences: "0101111101111100001111".

- **Phase 3**: Signal collection, 10-min duration. Running two pumps Sol, pump ThL, and pump Amp to ensure the reception of all transmitted bits.

The bit interval T_b remained fixed at 6.25 min, and the following values of α were used:

- $\alpha = 0.6$: The signal Y and the suppressor P were injected for a duration $\alpha T_\mathrm{b} =$ 3.75 min, with a guard interval of 2.5 min until the next bit.

- $\alpha = 0.92$: The signal Y and the suppressor P were injected for a duration $\alpha T_\mathrm{b} =$ 5.75 min, with a guard interval of 30 s until the next bit.

- $\alpha = 1$: The signal Y was injected during the whole bit interval while the suppressor P was not injected.

The decoded bit sequences for different α are illustrated in Supplementary Figure 11, and the corresponding typical bit errors are shown in Supplementary Figure 12.

Solution			Y	P	Sol Y	Sol P	ThL	Amp
pH			12.5	1.5	7.58	7.58	1.6	5.3
Flow rate (µL min^{-1})	Phase 1: Cleaning & baseline		0	0	192	192	128	128
	Phase 2&3: Bit transmission	Bit-0	0	0	192	192	128	128
		Bit-1 duty cycle	96	96	96	96	128	128
		Bit-1 guard interval	0	0	192	192	128	128
	Phase 4: Signal collection		0	0	192	192	128	128

Supplementary Table 8: **Bit Error Rate measurement.** Composition and injection flow rate of the different solutions injected for the experiment in Fig. 6a. To output a constant flow rate at the transmitter during the whole experiment, the flow rates of the two Sol pumps follow $Q_{\text{SolY}}(t) = 192 - Q_{\text{Y}}(t)$ and $Q_{\text{SolP}}(t) = 192 - Q_{\text{P}}(t)$, respectively.

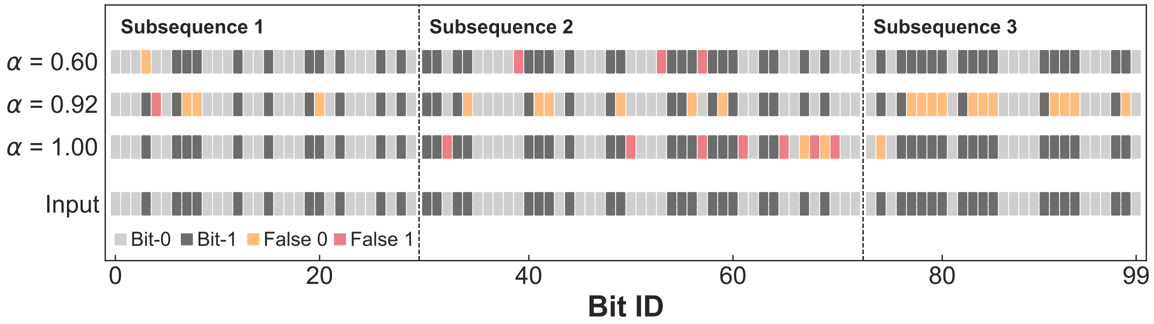

Supplementary Figure 11: **Decoded bit sequences**. Illustration of the complete input and decoded bit sequences to measure the BER (Fig. 6a), with the three subsequences illustrated one after the other. Each individual bit is represented by a rectangle, and the value of each bit is illustrated by the color of the band on the graph, with light and dark grey indicating successful transmission of bit-0 and bit-1, respectively. Orange indicates the detection of a bit-1 as a bit-0, and pink indicates the detection of a bit-0 as a bit-1.

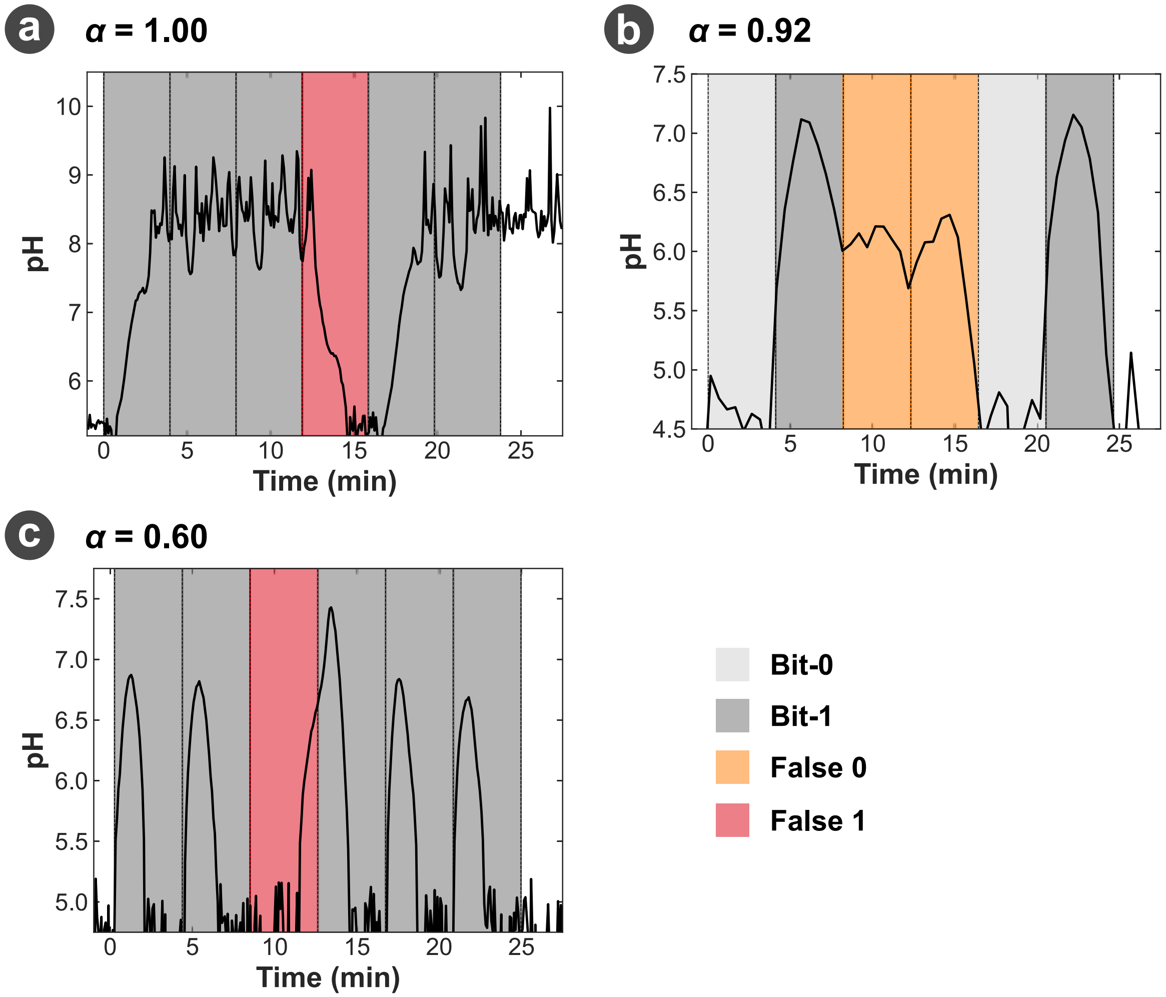

Supplementary Figure 12: **Typical bit errors.** Illustration of the typical bit errors observed during the BER measurements (Fig. 6a and Supplementary Figure 11) for various duty cycles α. For each setting, a 6-bit sequence is presented with the bit error at the center of the sequence, with **(a)** $\alpha = 1.00$ starting at bit #54, **(b)** $\alpha = 0.92$ starting at bit #40, and **(c)** $\alpha = 0.60$ starting at bit #55. The value of each bit is illustrated by the color of the band on the graph, with light and dark grey indicating successful transmission of bit-0 and bit-1, respectively. Orange indicates the detection of a bit-1 as a bit-0, and pink indicates the detection of a bit-0 as a bit-1.

6.9 Bit interval optimisation (Figs. 6c, d)

Experiment description. Measurement of the distortion appearing at high α values, optimisation of the bit interval.

Experimental setup. Full 5-pump version of the MIMIC platform, as illustrated in Fig. 1a. Distance between the transmitter and the receiver: 2 m. Distance between the final T-junction at the receiver and the UV-Vis spectrometer: 2 m.

Solution injection. The experiment was conducted in 3 phases, and the solutions' pH, solution injection, and flow rates are summarized in Supplementary Table 9.

- **Phase 1:** Cleaning and baseline, 30-min duration. Running two pumps Sol, pump ThL, and pump Amp to remove signal Y from the platform and provide a baseline for the experiment.
- **Phase 2:** Signal transmission. Two bit-1 are transmitted.
- **Phase 3:** Signal collection, 60-min duration. Running two pumps Sol, pump ThL, and pump Amp to ensure the reception of all transmitted bits.

The bit interval T_b varied from 1.25 to 90 min, while αT_b kept constant to 20 min.

Solution			Y	P	Sol Y	Sol P	ThL	Amp
pH			12.5	1.5	7.58	7.58	1.6	5.3
Flow rate (µL min^{-1})	Phase 1: Cleaning & baseline		0	0	24	24	16	16
	Phase 2: Bit transmission	Bit-1 duty cycle	12	12	12	12	16	16
		Bit-1 guard interval	0	0	24	24	16	16
	Phase 3: Signal collection		0	0	24	24	16	16

Supplementary Table 9: **Bit interval optimisation.** Composition and injection flow rate of the different solutions injected for the experiment in Figs. 6c, d. To output a constant flow rate at the transmitter during the whole experiment, the flow rates of the two Sol pumps follow $Q_\mathrm{SolY}(t) = 24 - Q_\mathrm{Y}(t)$ and $Q_\mathrm{SolP}(t) = 24 - Q_\mathrm{P}(t)$, respectively.

Supplementary figure

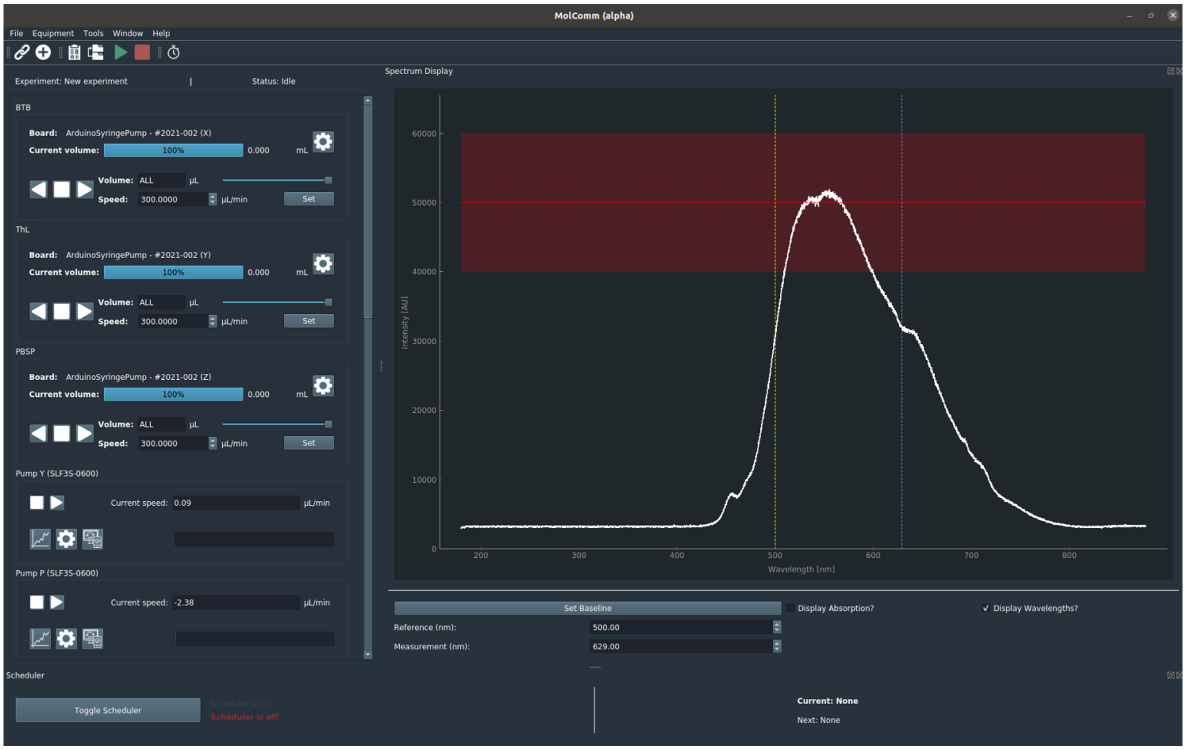

Supplementary Figure 13: **MolCommUI.** Screenshot of the Graphic User Interfaced Python software developed in this work to control and automate the MIMIC platform.

Supplementary references

[1] Elsbeth Klotz, Robert Doyle, Erin Gross, and Bruce Mattson. The equilibrium constant for bromothymol blue: A general chemistry laboratory experiment using spectroscopy. *Journal of Chemical Education*, 88:637–9, 2011.

[2] R W Sabnis. *Handbook of Acid-Base Indicators*. CRC Press, Boca Raton, Florida, 2007.

[3] Willem H Hundsdorfer, Jan G Verwer, and WH Hundsdorfer. *Numerical solution of time-dependent advection-diffusion-reaction equations*, volume 33. Springer, Berlin, Heidelberg, 2003.
